# Enhancing the analytic utility of clinical trial data to inform health disparities research

**DOI:** 10.1016/j.conctc.2020.100677

**Published:** 2020-11-25

**Authors:** Steven B. Cohen, Jennifer Unangst, Feng Yu

**Affiliations:** Division for Statistical and Data Sciences, RTI International, Washington, DC, USA

**Keywords:** Clinical trials, Health disparities, Project data sphere, Data integration, MEPS

## Abstract

Clinical trials are often conducted among younger, healthier, and less racially diverse patient populations than the population at large. Health disparities for individuals with cancer are most apparent when there are notable differences in the occurrence, frequency, burden of cancer and mortality rates among specific population groups. Enhancing the diversity of participants in clinical trials to reflect the characteristics of cancer survivors in the U.S. population is of growing interest to better insure the safety and efficacy of resultant treatments. The ***Project Data Sphere***® (PDS) cancer research platform is a first-of-its kind research environment that provides the research community with broad access to both de-identified patient-level clinical trial data and advanced analytic tools to enable big data-driven research. To address these analytic constraints, the data profiles in selected PDS patient-level cancer phase III clinical datasets have been augmented by linking the social, economic, and health-related characteristics of like cancer survivors from nationally representative health and health care-related survey data from the Medical Expenditure Panel Survey (MEPS). Our article shines a spotlight on this ongoing initiative to improve access to clinical trial data in support of health care disparities research initiatives.

Health disparities for individuals with cancer are most apparent when there are notable differences in the occurrence, frequency, burden of cancer and mortality rates among specific population groups; these differences often are manifest when comparing the experiences of distinct racial and ethnic minority groups. While research and policy efforts have helped to reduce some observed gaps in health outcomes, cancer disparities persist [1]. The driving factors for the continuance of disparities include differential access to and quality of care [2]. Clinical trials, for example, are used to identify safe and effective treatments for all those with cancer but are often conducted among younger, healthier, and less racially diverse patients than the population at large [3]. As a result, there is an increasing interest in diversifying clinical trial patients to ensure that resultant treatments are suited for those who are disproportionately affected in the first place. As a result, there is an increasing interest in diversifying clinical trial patients to ensure that resultant treatments are suited for subgroups who are underrepresented in trials and disproportionately affected by cancer.

As noted in a recent JAMA Oncology Viewpoint article, “data sharing in clinical trials is increasingly recognized as fundamental to strengthening therapeutic research [4].^”^ To this end, the ***Project Data Sphere***® (PDS) online platform is a centralized place where the cancer research community can broadly share, integrate, and analyze historical patient-level data from academic and industry phase III clinical trials. A primary goal of PDS is to unleash the full potential of existing clinical trial data and advance new research efforts that will improve the lives of cancer patients and their families around the world [5]. While PDS data are rich in measures that characterize the clinical trials under study, data providers are required to de-identify patient-level data by removing key social and demographic content that could otherwise be used to study underserved populations and the complex social, behavioral, and biological factors that contribute to inequities. To address these analytic constraints, with support provided by the Robert Wood Johnson Foundation, PDS and RTI International are collaborating to enhance the analytical utility of selected PDS datasets (downloadable from www.ProjectDataSphere.org). The effort has augmented the data profiles of cancer patients in selected PDS clinical trial datasets with social, economic, and health-related content from the nationally representative Medical Expenditure Panel Survey (MEPS). Patients from a representative set of PDS clinical trials were statistically linked with similar cancer survivors from MEPS to append measures of health care access and utilization, patient behaviors and attitudes toward care, and health conditions. This collection of content-enhanced PDS resources permit researchers to evaluate the efficacy of treatment-vs.-control randomizations, conduct probabilistic assessments of the representativeness of the cancer patients in these trials, and identify health disparities impacting on health outcomes. This initiative has been advanced to achieve the following objectives.

• To broaden the analytic capacity of PDS clinical trial data in support of health disparities and health outcomes research for cancer patients;
• To significantly scale up the analytic utility and content that can be realized by these data integration efforts;
• To conduct a broad array of assessments that investigate the representativeness of cancer clinical trial patients relative to characteristics of cancer survivors in the U.S. general population.

These data integration efforts linking the PDS-MEPS data resources also enable more targeted analyses that examine questions such as: How do disparities in cancer patients’ access to health care and income impact patient outcomes in specific phase III clinical trials? What variations in patient outcomes are associated with specific demographic, socioeconomic, and health-related factors?

*Project Data Sphere, LLC* (PDS), an independent, not-for-profit initiative of the *CEO Roundtable on Cancer's Life Sciences Consortium* (LSC), operates the *Project Data Sphere* platform, a free digital library-laboratory where the research community can broadly share, integrate and analyze historical, patient-level data from academic and industry phase III cancer clinical trials. PDS hosts over 200 phase III oncology clinical trial datasets, representing more than 150,000 cancer patients. Charter data providers include AstraZeneca, Bayer, Celgene, Janssen, Memorial Sloan Kettering Cancer Center, Pfizer, and Sanofi. This initiative extends the utility of these data by joining PDS patient-level data with nationally representative health-related data from the Medical Expenditure Panel Survey (MEPS). MEPS, sponsored by the Agency for Healthcare Research and Quality (AHRQ) is the nation's primary source of nationally representative, comprehensive, person-level data on health care use, insurance coverage, and expenses. Over the past several years, the MEPS data have supported a highly visible set of descriptive and behavioral analyses of the U.S. health care system [6].

Using data integration methods, sociodemographic, access, health, and health care-related measures associated with a nationally representative set of cancer survivors from MEPS are linked to similar cancer patients in the PDS analytic datasets using variables available in both data sources -- demographic information (age, race/ethnicity, and sex) and the EQ-5D™ index score, derived from the EuroQoL five-dimensions questionnaire. The EQ-5D™ has been administered in past implementations of MEPS, along with the 12-Item Short Form Health Survey (SF-12) developed from the Rand Medical Outcomes Study. The SF-12 is a general health status instrument with 12 questions producing two summary scores, the Physical Component Summary (PCS-12) and the Mental Component Summary (MCS-12). These scores are determined for each adult sample participant in MEPS based on their responses to the SF-12. These respective components are scored such that higher scores represent better physical and emotional function, and are standardized whereby the mean score is 50 and standard deviation is 10 in the general population. Using MEPS responses from the SF-12, predicted values of the EQ-5D™ index scores can be derived from MEPS for the years the EQ-5D™ instrument was not administered using an algorithm that only requires the availability of the MCS-12 and PCS-12 scores [7]. The prediction model follows: EQ-5D™ = 0.057867 + 0.010367·(PCS-12) + 0.00822·(MCS-12) - 0.000034·(PCS-12·MCS-12) - 0.01067.

The statistical linkage process uses a set of discriminatory variables that includes age, race, and sex, and the EQ-5D™ index score. Multiple datasets hosted on PDS include the EQ-5D™. The EQ-5D™ score is calculated directly for MEPS years where the EQ-5D™ questionnaire was administrated and is predicted for years when it was not. When additional demographic measures are available in both datasets (e.g., height, weight, body-mass index, employment status), they are also incorporated in the linkage process.

The MEPS typically surveys 2000 participating sample adults aged 18 and older with a reported cancer diagnosis. Several years of MEPS data on cancer survivors may be pooled to enhance the sample sizes of cases available for specific cancer classifications; this results in a much larger set of survivors of various cancer types available for linkage. The MEPS data files are accessible for downloading at the MEPS website: https://meps.ahrq.gov/mepsweb/data_stats/download_data_files.jsp. An examination of the demographic composition of distinct sets of cancer patients in the data enhanced PDS trials revealed significant departures from their representation in the nation. Cancer patients in the trials were often more likely to be younger, white, and male in contrast to the representation of cancer survivors in the United States. Cancer patients in the PDS trials were also significantly more likely to have better health states and also less likely to have chronic conditions such as hypertension, diabetes, asthma, arthritis, or coronary heart disease relative to the profiles of cancer survivors in the nation [8].

While cancer researchers continue to advance new discoveries and treatment protocols, millions of lives continue to be lost to cancer each year. The pace of progress in improving health outcomes in cancer patients is further challenged when addressing health disparities that impact specific populations such as racial minorities and economically disadvantaged population subgroups. Health disparities for individuals with cancer are most apparent when there are notable differences in the occurrence, frequency, burden of cancer and mortality rates among specific population groups. The analytically enhanced integrated data will help researchers explore the influence of healthcare access, socioeconomic factors, and health behaviors on the patient-level representativeness and outcomes data contained in the trials included in the PDS data enclave. Researchers can now access the data and supporting documents at https://data.projectdatasphere.org/projectdatasphere/html/landing/rti. As additional clinical trial datasets are added to the PDS website, researchers can also initiate future data augmentations using MEPS by implementing the delineated linkage methodology. This project further enables researchers to use the content enriched PDS datasets to stimulate new research findings and generate insights into the representational disparities that exist in trial study designs, thus helping to improve future study designs and to help promote equity in cancer research.

## Funding

This research was made possible through a grant from the 10.13039/100000867Robert Wood Johnson Foundation. Grant # 77003. The views expressed here do not necessarily reflect the views of the Foundation.
